# Supplementary material for: Daptomycin forms a stable complex with phosphatidylglycerol for selective uptake to bacterial membrane
Source: eLife. 2025 Jun 2;13:RP93267. doi: 10.7554/eLife.93267 (PMC12129450; doi:10.7554/eLife.93267)
Supplement: Supplementary file 1. [file elife-93267-supp1.docx]

**SupplementaryTable.** Assignment of the COSY signals of Dap in the fingerprint region.

| **Fingerprint COSY signals** | **Single Amino acid representation** | **Amino acid residues** |
| --- | --- | --- |
| 8.39 | U 13 | Kyn-13 |
| 8.32 | D 9 | Asp-9 |
| 8.20 | G 10, D 7 | Gly-10, Gly-5 |
| 8.16 | O 6 | Orn-6 |
| 8.14 | D 3 | Asp-3 |
| ?? Not present? | E 12 | 3mGlu-12 |
| 8.13 | N 2 | Asn 2 |
| 8.05 | A 8 | D-Ala-8 |
| 7.99 | T 4 | Thr-4 |
| 7.96 | S 11 | D-Ser-11 |
| 7.98 | ? | ? |
| 7.87 | W 1 | Trp-1 |
